# Supplementary figures and images for: Field Trials of Live and Inactivated Camelpox Vaccines in Kazakhstan
Source: Vaccines (Basel). 2024 Jun 19;12(6):685. doi: 10.3390/vaccines12060685 (PMC11209348; doi:10.3390/vaccines12060685)

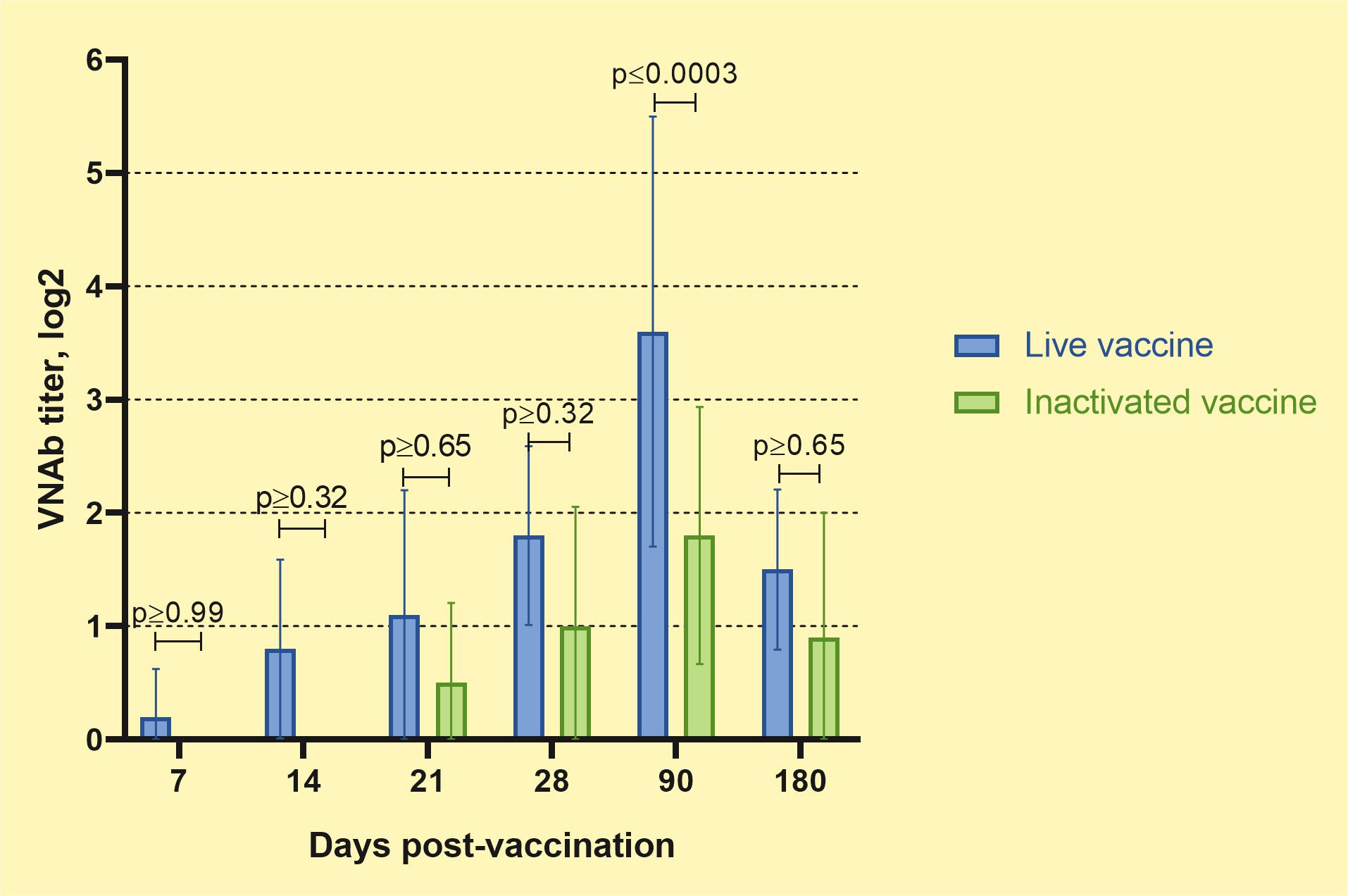

Supplement: Supplementary file 1 [file vaccines-12-00685-s001.zip › Suplementary Figure S1.jpg]
